# Neural Mechanisms of Reward Prediction Error in Autism Spectrum Disorder

**DOI:** 10.1155/2019/5469191

**Published:** 2019-07-01

**Authors:** Maya G. Mosner, R. Edward McLaurin, Jessica L. Kinard, Shabnam Hakimi, Jacob Parelman, Jasmine S. Shah, Joshua Bizzell, Rachel K. Greene, Paul M. Cernasov, Erin Walsh, Merideth A. Addicott, Tory Eisenlohr-Moul, R. McKell Carter, Gabriel S. Dichter

**Affiliations:** ^1^Department of Psychology and Neuroscience, University of North Carolina-Chapel Hill, Chapel Hill, NC 27514, USA; ^2^Duke-UNC Brain Imaging and Analysis Center, Duke University Medical Center, Durham, NC 27705, USA; ^3^Carolina Institute for Developmental Disabilities, University of North Carolina at Chapel Hill School of Medicine, Chapel Hill, Chapel Hill, NC 27510, USA; ^4^Center for Cognitive Neuroscience, Duke University Medical Center, NC 27705, USA; ^5^Department of Psychology and Neuroscience, University of Colorado Boulder, Boulder, CO 80309, USA; ^6^Department of Psychiatry, University of North Carolina-Chapel Hill, Chapel Hill, NC 57514, USA; ^7^Center for Addiction Research, University of Arkansas for Medical Science, Little Rock, AR 72205, USA; ^8^Department of Psychiatry, University of Illinois at Chicago, Neuropsychiatric Institute, Chicago, IL 60612, USA

## Abstract

Few studies have explored neural mechanisms of reward learning in ASD despite evidence of behavioral impairments of predictive abilities in ASD. To investigate the neural correlates of reward prediction errors in ASD, 16 adults with ASD and 14 typically developing controls performed a prediction error task during fMRI scanning. Results revealed greater activation in the ASD group in the left paracingulate gyrus during signed prediction errors and the left insula and right frontal pole during thresholded unsigned prediction errors. Findings support atypical neural processing of reward prediction errors in ASD in frontostriatal regions critical for prediction coding and reward learning. Results provide a neural basis for impairments in reward learning that may contribute to traits common in ASD (e.g., intolerance of unpredictability).

## 1. Introduction

Recent conceptualizations of autism spectrum disorder (ASD) have examined the disorder from the perspective of reward processing deficits, focusing primarily on social motivation and pleasure in the context of social and nonsocial rewards [1, 2]. From this framework, core deficits in social communication and interactions that characterize individuals with ASD may reflect or be caused by, at least in part, decreased motivation to engage with the social world, decreased feelings of pleasure during social interactions, and increased motivation for certain nonsocial rewards. In support of this model, individuals with ASD demonstrate atypical behavioral and neural responses to a range of rewards [3, 4].

Reward processing has been described in detail in a number of reviews [5, 6] and preclinical studies have delineated several reward processing dimensions, including (1) anticipation of reward, which includes approach and motivated behaviors; (2) receipt of reward, which encompasses hedonic response to rewards; and (3) reward learning, which refers to representations and predictions about future rewards [7]. Whereas most research into reward processing deficits in ASD has focused on responses during reward anticipation and receipt, neural mechanisms of reward learning are a critical feature of ASD that have not been fully addressed. This is a critical omission given that a number of theories suggest that ASD is characterized by impaired flexible responses to environmental contingencies [8] as well as impaired learning more generally (e.g., [9, 10]). The current study aimed to address this gap by examining the neural mechanisms of reward prediction errors (RPEs) in ASD.

Individuals with ASD typically demonstrate rigid patterns of thinking [11] that may impact their ability to learn from or draw upon past social interactions, even positive ones, to influence future behavior. Critically, impairment in the capacity to make reward-related associations is an important predictor of the development of social communication deficits in children with ASD [12, 13]. Functional neuroimaging studies have revealed decreased frontostriatal activity, specifically in the anterior cingulate cortex (ACC), ventral prefrontal cortex (vPFC), and striatum during implicit and explicit social (e.g., smiling faces, thumbs-up) reward learning tasks in children and adults with ASD [14, 15]. Additionally, increased activation in the ACC, superior and middle frontal gyri, and putamen during social implicit learning has been observed in ASD [16, 17]. Taken together, these findings indicate altered neural processing during reward learning in ASD.

Although there is a growing body of literature addressing reward learning in ASD, relatively fewer ASD studies have directly examined RPEs specifically. RPEs occur when there is a mismatch between an expected and a received outcome [18] and have a powerful influence on reward learning and subsequent behavior. If a reward-related outcome is exactly as predicted (i.e., no prediction error), a cue-reward association is maintained, and the subsequent behavior remains unchanged. However, if a reward-related outcome is more or less valuable than predicted, such that a positive or negative prediction error occurs, the association between stimulus or action and reward can be modified to better learn the former's predictive value.

There is evidence that behavioral and cognitive inflexibility and rigidity in ASD may result from atypical computation of prediction errors [8, 19, 20]. Certain ASD traits, such as ritualistic behaviors and insistence on sameness, may reflect behaviors that function to minimize environmental unpredictability. This is supported by evidence that some individuals with ASD have adverse reactions to unexpected, unpredictable events [21, 22]. Additionally, children with ASD may prefer to engage in predictable or repetitive tasks [23]. Supporting the linkage between impaired neural responses to RPEs and ASD symptom severity, Balsters et al. [24] reported reduced neural responses in the ACC in ASD during social prediction errors, and the degree to which these responses were aberrant correlated with overall ASD symptom severity.

The goal of the present study was to assess neural responses during a prediction error task in ASD. Modeling after work by Ramnani and colleagues [25] and Addicott and colleagues [26], we assessed neural responses to reward-related prediction errors when monetary rewards were delivered independently of goal-directed actions. Participants completed a prediction error task during functional magnetic resonance imaging (fMRI). Many cognitive neuroscience learning studies have examined neural correlates associated with receiving rewards; the omission of an expected reward is mediated by similar neural circuitry [27, 28], and there is evidence to suggest that the omission of an expected reward may also be a powerful input to this system [29]. Thus, this study examined neural responses to unexpected reward and unexpected nonreward prediction errors, as well as to expected and unexpected rewards.

Preclinical findings strongly implicate the dorsal and ventral striatum (VS) in processing RPEs [30], and human neuroimaging studies similarly reveal RPE signals in reward-related structures including the VS as well as frontotemporal circuits [31]. A recent meta-analysis implicated multiple frontostriatal regions, particularly frontal gyri, the ACC, and the insula (for a review see Garrison, Erdeniz, and Done (2013), [32]). Considering these findings and the literature outlining altered reward learning in ASD (e.g., [14, 24]), we hypothesized that the ASD group would be characterized by decreased activation of striatal and prefrontal cortical regions during RPEs. We further explored relations between neural response to RPEs, behavioral task responses, and ASD symptom severity in the ASD group.

## 2. Methods

### 2.1. Participants

This protocol was approved by the Institutional Review Boards at the University of North Carolina at Chapel Hill and Duke University Medical Center. All procedures were in accordance with the ethical standards of the institutional and/or national research committee and with the 1964 Helsinki declaration and its later amendments or comparable ethical standards. Prior to participation, informed consent was obtained from all participants.

Participants with ASD were recruited through the Autism Research Subject Registry maintained through the Carolina Institute for Developmental Disabilities as well as the Autism Society of North Carolina and Autism Speaks. Typically developing controls (TDCs) were recruited via lists maintained by the Duke-UNC Brain Imaging and Analysis Center (BIAC). Participants with ASD were high functioning, defined as having fluent phrase speech and nonverbal IQ>70. Exclusion criteria for both groups included known sensory deficits or medical conditions, history of neurological injury (i.e., head trauma, poorly controlled seizure disorder (seizure within the preceding six months), stroke, prior neurosurgery, or being under the care of a neurologist or neurosurgeon as determined by interview, diagnosis of intellectual disability, and MRI contraindications.

Sixteen adults with ASD and 16 TDCs, 18-53 years old, participated. ASD diagnoses were based on a history of clinical diagnosis confirmed via Module 4 of the Autism Diagnostic Observation Schedule, Second Edition (ADOS-2; [33]; Lord et al., 2012), administered by a research reliable assessor and using standard algorithm cutoffs for ASD. Additionally, autism symptoms were assessed in the ASD group via the Social Responsiveness Scale (SRS), a self-report instrument that provides a dimensional measure of ASD impairments [34]. Prior to study enrollment, typically developing adults completed a brief screener for cognitive functioning (measured via the North American Adult Reading Test (NAART) [35]) to ensure comparability with the ASD group. Potential control participants with scores greater than 120 on verbal and performance intelligence quotient (IQ) scores were excluded from the study. Of the 32 participants enrolled, data from 30 were analyzable: two TDC participants did not complete the MRI scan due to discomfort in the scanner. Therefore, the final sample included 16 adults with ASD and 14 TDCs (Table 1 provides participant demographic information). Of the final sample, groups did not differ in full-scale IQ (measured via the Wechsler Abbreviated Scale of Intelligence (WASI; [36])) or gender distribution (*p*'s>.05). Groups did differ in age,* t*(29)=4.85,* p*<.0001, reflecting younger individuals in the ASD group (*M*=19.50,* SD*=2.07) relative to the TDC group (*M*=31.57,* SD*=9.99).

### 2.2. Materials and Measures

#### 2.2.1. fMRI Task

The functional magnetic resonance imaging (fMRI) task was a prediction error task, a computerized task of learned outcome expectancies (modeled after Ramnani et al. [25] and Addicott, Oliver, and Joseph McClernon [26]). Each task trial consisted of two phases: a cue phase and an outcome phase. The cues (cue A and cue B) were represented by quilt squares, and the outcomes (reward and nonreward) were represented by the image of a $100 bill and a blurry rectangle, respectively. During each trial, a cue was presented for 2 seconds during which the participant guessed whether the cue predicted a reward or a nonreward by pressing response buttons as quickly as possible corresponding to “$” and “0” shown on screen. The location of the “$” and “0” on screen (left or right) was counterbalanced across participants. After responding to the cue, a box outlined the selection and the outcome was shown for 1 second, indicating whether the outcome was correctly predicted; however, participant responses did not affect outcomes. If no response was made, “Missed Response” was shown during the outcome phase and that trial was excluded from analyses. Between the cue and outcome was a jittered delay from 0.8 to 1.6 seconds. Intertrial intervals ranged from 1.5 to 6 seconds with a positively skewed distribution.

During the practice session, participants completed an 80-trial training version of the task. In this version, one cue was always rewarded, and the other cue was not (counterbalanced across participants). To ensure task comprehension and reward learning, participants achieved at least 90% accuracy on this training version before continuing with the fMRI portion of the study. In this way, the task was specifically designed to ensure performance did not significantly differ between the ASD group and TDC group in terms of accuracy and reaction time [25, 37].

During the MRI scan, participants completed two task runs. Each run consisted of 100 trials: 50 with cue A and 50 with cue B. In 80% of the trials, the cue-outcome relationship was the same as in the training version (i.e., no prediction errors); however, in 20% of the trials, the cue-outcome relationship was the opposite to the training version (i.e., prediction errors). This resulted in four outcome conditions: expected reward, expected nonreward, unexpected reward, and unexpected nonreward. The first 11 trials in the scanner did not produce any prediction errors to allow for responses to reinforce the cue-outcome pairings prior to eliciting prediction errors. Participants were told that every time they saw a $100 bill (i.e., during a reward outcome trial), they would get points that went towards a $10 bonus. However, points would be deducted for every response they missed. At the end of the scan, participants were awarded a $20 bonus in addition to the study compensation.

### 2.3. Procedure

Participants in the ASD group completed the study across two separate visits while those in the TDC group completed the study in one visit. For the ASD group, the first visit included diagnostic (ADOS-2), cognitive (WASI), and symptom (SRS) assessments. For both groups, visit two included the practice scanner task and the MRI scan. For the TDC group, visit two (the only visit for this group) also included completing the WASI. ASD participants received a base rate of $10, plus $10 per hour for the first 2-4-hour testing session. During the second visit for ASD participants and the visit for TDC participants, participants received a base rate of $15 for procedures outside of the scanner, plus $20 per hour for the 1.5-hour scan in addition to the previously mentioned $20 bonus.

### 2.4. MRI Data Acquisition and Preprocessing

MRI data were acquired at the Duke-UNC Brain Imaging and Analysis Center (BIAC) on a General Electric (Waukesha, WI) MR750 3.0T scanner equipped with 50 mT/m gradients and an eight-channel head coil for parallel imaging. High-resolution T1-weighted anatomical images were acquired with 162 axial slices using a FSPGR pulse sequence (TR = 8.16 ms; TE = 3.18 ms; FOV = 256 mm; image matrix = 256 × 256; voxel size = 1 × 1 × 1 mm; flip angle = 12°) used for normalization and coregistration. This structural image was aligned in a near axial plane defined by the anterior and posterior commissures. Whole-brain functional images were acquired using a spiral-in SENSE sequence (TR = 1580 ms; TE = 30 ms; FOV = 240 mm; image matrix, 64 × 64; flip angle = 60°; voxel size, 3.75 × 3.75 × 3.80 mm; 37 axial slices). The first four volumes of each functional run were discarded to allow for steady state equilibrium.

Functional data were preprocessed using FSL version 5.0.1 (Oxford Centre for Functional Magnetic Resonance Imaging of the Brain (FMRIB), Oxford University, UK). Preprocessing was applied in the following steps: (i) brain extraction for non-brain removal [38]; (ii) motion correction using MCFLIRT [39] as well as motion correction, “Standard+Extended Motion Parameters” option within FSL expert analysis tool (FEAT), which includes the six head motion parameters as estimated by MCFLIRT as confound regressors as well as an additional 18 motion regressors (derivatives of the original six head motion parameters, the squares of these derivatives, and the original six motion parameters); (iii) spatial smoothing using a Gaussian kernel of FWHM 5 mm; (iv) mean-based intensity normalization of all volumes by the same factor; and (v) high-pass filtering [40]. Functional images of each participant were coregistered to structural images in native space, and structural images were normalized into a standard stereotaxic space (Montreal Neurological Institute). Registrations used an intermodal registration tool [38, 40]. Voxel-wise temporal autocorrelation was estimated and corrected using FMRIB's Improved Linear Model [38, 41].

### 2.5. fMRI Data Analyses

Key anatomical regions within the reward system (frontal lobes, amygdala, nucleus accumbens, insula, thalamus, caudate nucleus, anterior cingulate gyrus, and putamen) that have previously been found to be functionally involved in reward learning as well as impaired in ASD [42, 43] (Schultz, 2015) were defined* a priori* for small volume correction. These regions were generated in FSL using the Harvard-Oxford cortical and subcortical structural probabilistic atlases. All masks were thresholded at 25%, binarized, and then combined into a single mask using fslmaths. For all analyses, voxels were considered significant if they passed a threshold of p<.002, uncorrected, and were part of a 32-voxel cluster of contiguous significant voxels, corresponding to a family-wise corrected p<.05. This cluster size was determined by using Monte Carlo simulations via the updated version 3dFWHMx and using 3dClustSim programs from AFNI software package [44]. Localizations were based on Harvard-Oxford cortical and subcortical structural probabilistic atlases as implemented in FSLView version 3.1.8, and all activation maps were visualized with MRIcron (https://www.nitrc.org/projects/mricron/).

We then conducted a general linear model (GLM) using FEAT to examine group differences with respect to contrasts of interest. First level analyses were conducted for each participant which included a total of six regressors, each of which was convolved with a double-gamma hemodynamic response function. Regressors one through three modeled the orthogonal components of the mean activation across all events from (1) reward cues, (2) nonreward cues, and (3) outcomes. We also included three regressors as parametric modulators of the outcome events that described changes relative to the mean of the outcome activation (regressors four through six). Regressor four, the signed prediction error [45], modeled positive changes for all unexpected rewards and negative changes for all unexpected reward omissions relative to the mean event activation. For a given trial (*t*) the signed prediction error (SPE) is the difference between the experienced reward on that trial (*r*_*t*_) and the expected reward for that stimulus (*E*[*r*]); see (1).

(1) $$\begin{array}{c} SPE=r_{t}-E\left[\;r\;\right] \end{array}$$

For regressor five, we employed a variant of the unsigned prediction error (e.g., Matsumoto & Hikosaka, 2009), contrasting expected and unexpected outcomes regardless of magnitude. Unexpected outcomes (large RPEs that were positive or negative) were modeled as positive deviations from the mean event activation, and expected outcomes (small RPEs that were positive or negative) were modeled as negative deviations (-1) (no trial outcomes had a prediction error of zero); see (2). Our outcomes contain only two levels of deviation from the mean reward, allowing only a two-level contrast and not a true parametric model. We have therefore labeled this regressor as the thresholded unsigned prediction error (tUPE).

(2) $$\begin{array}{c} tUPE=\left\{\begin{array}{cc} +1, & -0.2>r_{t}>0.2\\ -1, & -0.2<r_{t}<0.2 \end{array}\right. \end{array}$$

Regressor six modeled the mean of all rewarded outcomes (+1) relative to unrewarded outcomes (-1), regardless of the magnitude of the outcome. This regressor is similar to (but not the same as) the SPE parametric regressor. Instead of modeling the magnitude of the reward surprise, it models the receipt of all rewards as producing the same response. Whereas it is not always included, we include it here to provide a point of comparison with previous work using the same task [26]. Each parametric outcome regressor was orthogonalized with respect to the mean outcome, and contrasts were defined for each outcome. Cue phase regressors were included to control for overall BOLD variance but are not the subject of the present hypotheses and thus are not presented.

A second-level fixed-effect analysis averaged the contrasts across the two functional runs for each participant. Finally, the primary method of analysis was to identify clusters that showed a main effect of group (ASD versus TDC). Group-level analyses covaried for age. Group-wise activation images were calculated by a mixed effects higher-level analysis using Bayesian estimation techniques with FMRIB Local Analysis of Mixed Effects (FLAME 1+2; [38, 46]).

## 3. Results

### 3.1. Motion

No participants had motion that was >2.5 mm along any of the six axes (i.e., x, y, z, pitch, yaw, and roll). There were also no group differences in any of the six motion parameters (*p*'s>.05).

### 3.2. Task Reaction Time and Accuracy

Groups did not differ in the percentage of missing trials,* p*>.05 (ASD* M*=1.38,* SD*=0.01; TDC* M*=1.64,* SD*=0.01). Analyses of reaction times (RTs) and task accuracy also revealed no group differences in RT and accuracy (*p*'s>.05).

### 3.3. Behavioral Indices of Learning during the fMRI Task

To address whether groups differed in learning behavior from choice data collected during scanning, we used multilevel logistic modeling to compare groups in terms of the effect of accuracy on a given trial (correct or incorrect) to accuracy on the subsequent trial. There was no significant difference between groups in this behavior, *γ*_PriorAccuracy(0/1)*∗*Group(ASD/CON)_ =.88, SE =.73, t(26) = 1.21,* p* = 0.24. This suggests that there were no group differences in the association between accuracy effect on the prior trial and accuracy effect on the current trial.

### 3.4. fMRI

**Figure 1:**
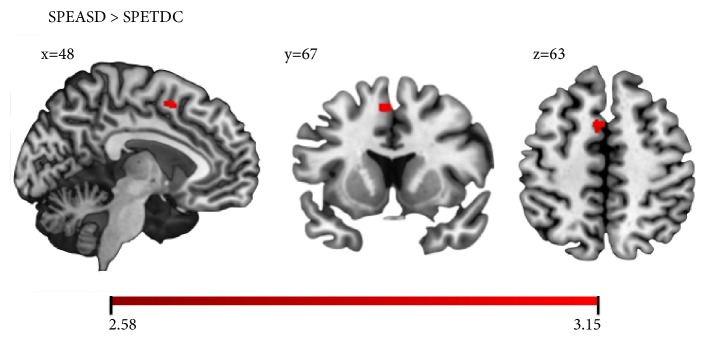
Greater activation in the ASD group relative to the TDC group in the left paracingulate gyrus during the signed prediction error (SPE) contrast. Color bar represents the range of z-values.

**Figure 2:**
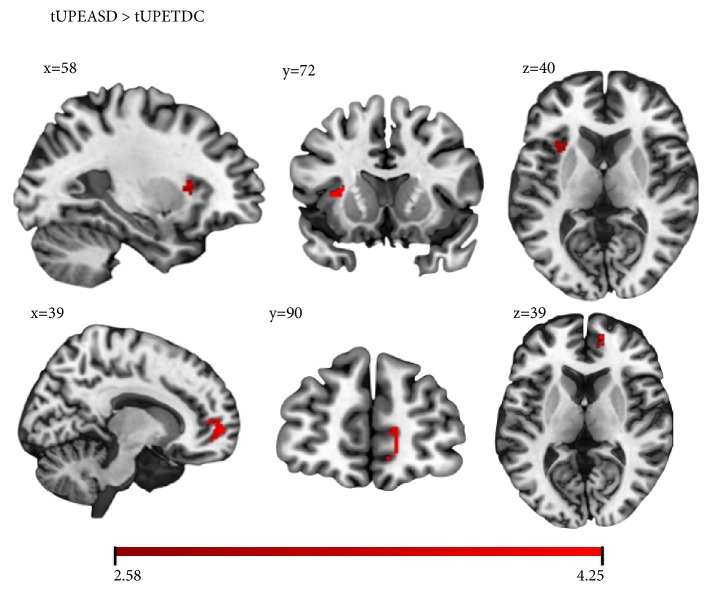
Greater activation in the ASD group relative to the TDC group in the left insula (top row) and in the right frontal pole (bottom row) during the threshold unsigned prediction error (UPE) contrast. Color bar represents the range of z-values.

With respect to the signed prediction error condition (SPE, (1)), there was greater activation in the ASD group relative to the TDC group in the left paracingulate gyrus (see Figure 1 and Table 2). With respect to the thresholded unsigned prediction error condition (tUPE, (2)), brain regions with greater activation in the ASD group relative to the TDC group included the left anterior insula and the right frontal pole (see Figure 2 and Table 2). Results revealed no group differences in activation with respect to the main effect of reward (reward outcomes > nonreward outcomes). There were no regions with greater activation in the TDC group relative to the ASD group for any of the contrasts examined.

#### 3.4.1. Whole-Brain Voxel-Wise Analyses

Results described above were not significant in the context of a corrected whole-brain voxel-wise analyses.

#### 3.4.2. fMRI Correlations with Task Performance and ASD Symptom Severity

Correlations were explored between task accuracy or RTs, SRS total t-scores, ADOS-2 calibrated severity scores [33, 47], and clusters that revealed group differences (i.e., the left paracingulate gyrus in the signed prediction error condition and the right frontal pole and the left insula in the thresholded unsigned prediction error condition). Results revealed no significant relationships in these clusters (*p*'s>.05).

## 4. Discussion

The goal of the present study was to examine neural responses to RPEs in ASD. We used a functional neuroimaging task designed to assess RPEs and hypothesized that, relative to the TDC group, individuals with ASD would demonstrate reduced frontostriatal activation during RPEs. This hypothesis was informed by patterns of behavioral and cognitive rigidity and inflexibility in ASD (e.g., [8, 24]). Contrary to hypotheses, we found greater activation in the left paracingulate gyrus in ASD during the signed prediction error condition. Results also revealed group differences in the thresholded unsigned prediction error condition, such that the ASD group showed relatively greater activation in the left insula and the right frontal pole relative to controls.

Despite the growing number of neuroimaging studies examining reward processing in ASD, few studies have examined RPEs in ASD: previous studies revealed hypoactivation and hyperactivation in frontostriatal regions (e.g., ACC, vPFC) in children and adults with ASD during implicit and explicit social learning tasks [14–16]. The current study adds to these findings by assessing neural mechanisms of RPEs, a fundamental component of reward learning that has not been examined to date in ASD. Our finding of hyperactivation in the insula and frontal pole in ASD to RPEs implicates regions that have been implicated in RPEs in nonclinical studies [32]. Our results also complement findings from Balsters et al. [24] who found reduced or absent neural responses in striatal regions in ASD during social prediction errors.

We did not find differential activation in the striatum in ASD. This is surprising given that preclinical studies strongly implicate dorsal and ventral aspects of the striatum in RPEs [30]. However, nonclinical neuroimaging studies have revealed variability in regions implicated in RPEs, with activation localized to multiple frontotemporal regions [32]. Indeed, the RPE task in the current study was modeled from the task reported by Ramnani et al. [25] wherein frontal pole activation to RPEs was reported. Thus, our localization of differential activation in the ASD group to the insular cortex and anterior and frontal pole during RPEs implicates regions previously linked to RPE processing in nonclinical samples.

Increased left anterior insula in response to RPEs in ASD implicates a brain region that has been shown to encode RPEs in neuroeconomic studies of processing uncertainty [32, 48–50]. Additionally, nonclinical studies report increased anterior insula signals in response to ambiguity [51] that contribute to feelings of uncertainty. The anterior insula has also been implicated in processing information about risk associated with decisions and mediating behavioral and physiological effects of risk prediction (e.g., [52–54]). The anterior insula has been further hypothesized to link affective processing with motivation, decision-making [54], and a variety of negatively and positively valenced emotional processes [55]. This region is also positioned anatomically to play a critical role in linking affective value with adaptive behavior [56, 57]. In sum, the capacity of the anterior insula to detect changes in bodily states and initiate motivated, reward-related adaptive behaviors highlights its critical role in RPEs.

Functional abnormalities in the anterior insula have been frequently reported in ASD across many task contexts [58]. A meta-analysis of functional neuroimaging studies in ASD revealed hypoactivation of the anterior insular cortex during tasks related to social and emotional processing [59]. Some studies reported mainly right-lateralized task-related anterior insular hypoactivation in ASD (e.g., [60, 61]; e.g., [62]) whereas others have reported bilateral anterior insula hyperactivation in ASD (e.g., [63–66]).

The current study also observed relative hyperactivation in ASD in the right frontal pole, a region implicated in higher-order cognitive operations, including decision-making and planning, including monitoring and evaluating reward- and punishment-based decisions [67]. The frontal pole additionally promotes learning cue associations related to costs and benefits, thereby impacting reward-based behaviors [68]. Human lesion studies suggest that regions of the frontopolar cortex may also be involved in orienting attention to future events [69], and Ramnani et al. [25] reported frontal pole activation in a nonclinical sample in response to RPEs. Aberrant activation of the left and right frontal pole in response to social and monetary reward anticipation and outcome has been reported in adults with ASD [42, 64], and Scott-Van Zeeland et al. [15] found greater activation of the frontal pole in children with ASD relative to typically developing children in response to monetary rewards using an implicit learning task. Given the role of this region for evaluating decisions and planning after reward presentation [67], differential activation of the frontal pole in ASD may indicate altered decision-monitoring behaviors in response to RPEs in ASD.

Our observed hyperactivation of the left anterior insula and right frontal pole in ASD provides evidence for atypical neural processing of RPEs in ASD in regions critical for prediction coding and reward-related behaviors. These findings provide a neural basis for ASD traits such as insistence on sameness that may reflect a heightened awareness, but decreased tolerance, for unpredictability [8, 19]. Additionally, intolerance of unpredictability may impact social communication in ASD given that social situations are highly unpredictable. For instance, theory-of-mind, a well-documented deficit in ASD, requires the capacity to predict the future behaviors of others based on past and current behaviors [70]. Taken together, altered neural response to RPEs suggests that the observed real-world reward learning impairments of individuals with ASD (e.g., [9, 10]) may be a reflection of difficulty responding adaptively when prediction errors occur and, thus, provides a neural basis for impaired reward learning behaviors in ASD (e.g., [13, 14, 16]). Findings also revealed group differences in the signed prediction error condition, such that the ASD group demonstrated relative hyperactivation of the paracingulate gyrus during unexpected outcomes relative to controls. Hyperactivation of the paracingulate gyrus has been previously linked to atypical nonsocial reward anticipation and outcomes in ASD [42], and the current study extends the pattern of reward-related paracingulate gyrus hyperactivation in ASD to the context of RPEs.

Neural responses to RPEs and the severity of ASD symptoms were not correlated. Although endophenotypic data such as fMRI may provide unique information relative to symptom measures, the ASD sample size in the present study attenuated power to detect such correlations. Future studies with larger samples will be needed to more definitively explore whether neural responses to RPEs correlate with ASD symptom severity. Additionally, future work with age-matched samples will be needed to assess the impact of age on RPEs in ASD.

### 4.1. Implications

The present findings suggest neural mechanisms for challenges tolerating unpredictability and insistence on sameness behaviors that are often observed in ASD [11]. Future fMRI studies that explore probabilistic reversal learning will be needed to determine whether aberrant neural response to RPEs spurs aberrant learning rates of new contingencies in ASD. The current findings also suggest a neural mechanism underlying impaired reward learning in ASD which may have important implications for ASD interventions. Many ASD interventions use a variety of rewards as motivators for learning with varying success [71–73]. It may be the case that a better understanding of RPEs in ASD may inform the development of ASD treatment strategies that use appropriate behavior-contingent rewards as motivators for learning social skills.

## Figures and Tables

**Table 1:** Participant characteristics (means and standard deviations).

	ASD (*n*=16)	TDC (*n*=14)	*p* value
Age	19.50 (2.07)	31.47 (9.99)	<.01
Male: Female Ratio	13:3	5:2	.53^†^
Full Scale IQ	106.93 (18.22)	110.33 (14.16)	.57
Verbal IQ	103.50 (21.29)	111.53 (11.80)	.22
Performance IQ	98.50 (29.12)	106.40 (14.40)	.46
ADOS-2 Total (SA +RRB) Score	16.13 (4.21)	--	
ADOS-2 Calibrated Severity Score	8.2 (1.42)	--	
SRS Total *t* score	71.81 (8.46)	--	

*Note.* ADOS-2: Autism Diagnostic Observation Schedule, 2^nd^ edition, SA: social affect, RRB: restricted and repetitive behaviors, SRS: Social Responsiveness Scale, †: Pearson's X^2^.

**Table 2:** Frontostriatal functional activation clusters showing ASD>TDC differences in voxel-wise analyses. No regions showed ASD<TDC differences (see text for details).

Condition	Region	Hem	k	BA	x	y	z	Z max
Signed Prediction Error	Paracingulate Gyrus	L	39	--	48	67	63	3.15

Threshold Unsigned Prediction Error	Frontal Pole	R	115	10	39	90	39	3.15
	Frontal Pole	L	61	9	63	84	50	3.08
	Frontal Pole	R	36	10	31	95	38	3.09
	Anterior Insula	L	105	--	58	72	40	4.25

*Note.* Hem: hemisphere; k: cluster size in voxels; BA: Brodmann area; Z max: maximum z-value.

## Data Availability

The neuroimaging data used to support the findings of this study are available from the corresponding author upon request.

## Supplementary Materials

Supplementary Materials Figure 1: Responses to signed prediction errors (SPEs, see (1) in the main text) for the control and ASD group separately. This figure shows that the control group demonstrated activation in the paracingulate gyrus, the right thalamus, and the right caudate, whereas the ASD group did not demonstrate striatal activation during prediction errors. Figure 2: responses to thresholded unsigned prediction errors (tUPE, see (2) in the main text), for the control and ASD group separately. This figure shows that the control group demonstrated activation in the dorsal and ventral striatum, whereas the ASD group did not demonstrate striatal activation during prediction errors.
