# Supplementary material for: Neural Mechanisms of Reward Prediction Error in Autism Spectrum Disorder
Source: Autism Res Treat. 2019 Jul 1;2019:5469191. doi: 10.1155/2019/5469191 (PMC6634058; doi:10.1155/2019/5469191)
Supplement: Supplementary Materials — Figure 1: Responses to signed prediction errors (SPEs, see (1) in the main text) for the control and ASD group separately. This figure shows that the control group demonstrated activation in the paracingulate gyrus, the right thalamus, and the right caudate, whereas the ASD group did not demonstrate striatal activation during prediction errors. Figure 2: responses to thresholded unsigned prediction errors (tUPE, see (2) in the main text), for the control and ASD group separately. This figure shows that the control group demonstrated activation in the dorsal and ventral striatum, whereas the ASD group did not demonstrate striatal activation during prediction errors. [file 5469191.f1.docx]

**Supplementary Materials for “Neural Mechanisms of Reward Prediction Error in Autism Spectrum Disorder”**

**Responses to signed prediction errors (SPEs, see eq. 1 in the main text) for the control and ASD group separately.** This figure shows that the control group demonstrated activation in the paracingulate gyrus, the right thalamus, and the right caudate, whereas the ASD group did not demonstrate striatal activation during prediction errors.


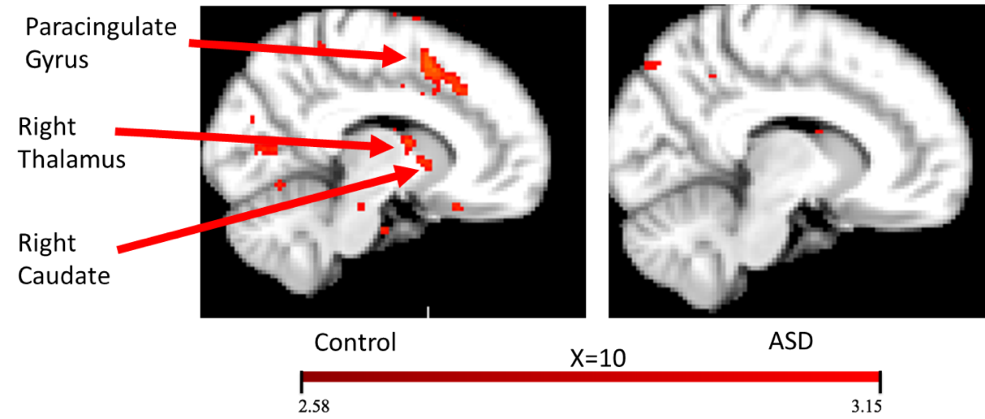


**Responses to thresholded unsigned prediction errors (tUPE, see eq. 2 in the main text), for the control and ASD group separately.** This figure shows that the control group demonstrated activation in the dorsal and ventral striatum, whereas the ASD group did not demonstrate striatal activation during prediction errors.

**
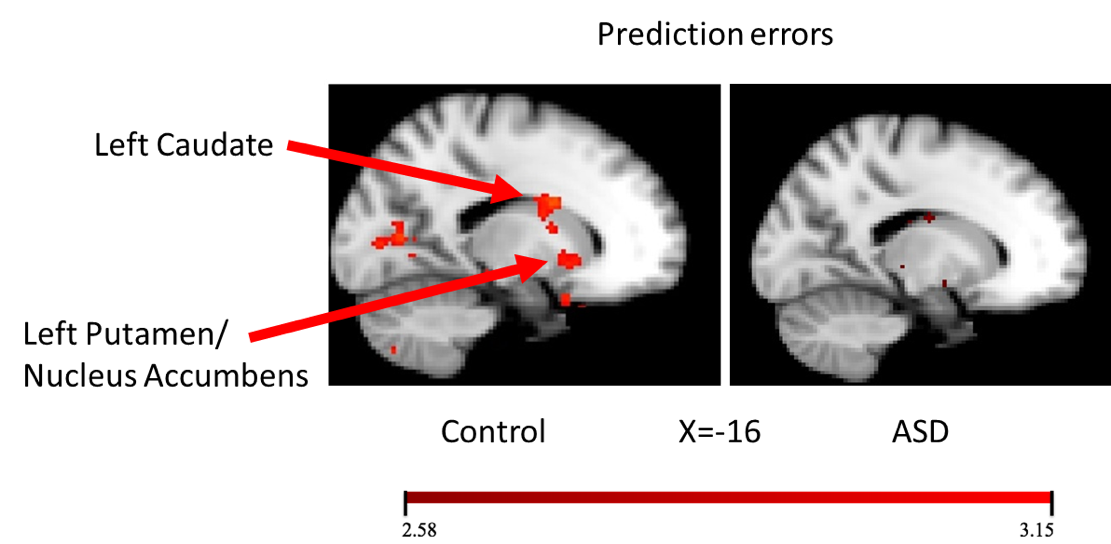
**
